# Physiological Chemistry

**Published:** 1899-04

**Authors:** John A. Miller


### Progress in Medical Science.

### PHYSIOLOGICAL CHEMISTRY.

BY JOHN A. MILLER, PH. D.

### ABSORPTION AND EXCRETION OF IRON IN THE HUMAN AND ANIMAL BODY.

A. HOFMANN, Zurich (*Virchow's Archiv.*, 1898; *J. Chem. Soc.*, 1898). Microchemical investigation of the wall of the alimentary canal shows that in man the principal absorption is in the duodenum. The appearances are little different when medicinal doses of iron are added to the food; the liver, and especially the spleen, are places where the iron is stored, whilst it is excreted by the kidney and large intestine. Analogous results were found in the guinea-pig. It is believed that inorganic salts of iron are capable of absorption.

### METABOLISM IN LEUCEMIA AND PSEUDOLEUCEMIA.

WACLAW VON MORACZEWSKI (*Virchow's Archiv.*, 1898; *J. Chem. Soc.*, 1898.) Complete tables are given of the analyses of food and excretions in a case of leucemia and in one of pseudoleucemia; the observations extended over a prolonged period. As a result, leucemia is characterised as a nitrogen and phosphorus disease; pseudoleucemia as a nitrogen disease. As in other forms of anemia, they may also be regarded as chlorine and calcium diseases, that is to say, there is a retention in the body of the substances mentioned; the katabolic side of metabolism is in abeyance and this is possibly connected with diminution of oxidation processes. Treatment with spleen tabloids had practically no effect; with oxygen, the excretion of phosphorus and calcium is increased; with thyroid tabloids, the metabolism becomes almost normal.

### ESTIMATION OF GLUCOSE IN URINE BY MEANS OF METHYLENE-BLUE.

GOFF (*J. Chem. Soc.*, 1898). Although urine decolorises methylene-blue, it has no such action if it has been previously strongly diluted. Urine may, therefore, be tested for glucose by adding to 1 c.c. of the diluted sample (1:3) 5 c.c. of a solution of methylene-blue (1:5000) containing a few drops of aqueous potash. Normal urine remains blue, but diabetic urine is at once decolorised or turns pale yellow. To render this reaction quantitative, 30 c.c. of methylene-blue solution mixed with 1 c.c. of 4.5 per cent. aqueous potash is added drop by drop to 1 c.c. of diluted urine, covered with a little xylene and placed in boiling water until there is a permanent blue coloration. The xylene serves to protect the solution from atmospheric oxygen. The sample of urine should not contain more than 0.3 per cent. of glucose. One c.c. of 0.1 per cent. solution of glucose requires 6.5 c.c. of the reagent. Urea, uric acid, sodium chloride, creatinine, peptones, albumin do not reduce the blue; biliary matters turn it green.

---

### CHEMISTRY AND ACTION OF THE THYROID GLAND.

ROBERT HUTCHISON (*J. Physiol.*, 1898; *J. Chem. Soc.*, 1898). The iodine contained in the colloid matter of the thyroid varies considerably and averages 0.309 per cent. Of the digestion product of the colloid, only those which contain iodine are active. Iodised nucloalbumin, made artificially from the thymus, has no physiological activity, and increase in activity of the thyroid colloid is not produced by artificial addition of iodine, although in the natural products the amount of iodine and the activity vary directly. Intravenous injection of the colloid has no effect on blood pressure, heart or coagulation of the blood. Thyroid extracts produce a fall of arterial pressure, but this is due to extractives and partly to mineral salts present.

Previous removal of ovaries or testes has no influence on the results of thyroidectomy and ovarian feeding has no curative influence in myxedema. No poison was discovered either in the bile or the central nervous system after thyroidectomy.

Of twenty-four consecutive cases of complete thyroidectomy, only four survived; this number can only be slightly increased by thyroid feeding. Para-thyroid feeding has no effect in myxedema. Keeping the animals warm after the operation has no effect.

### CHANGES IN THE URINE PRODUCED BY EXERCISE AND BY TURKISH BATHS.

G. C. Garratt (*J. Physiol.*, 1898; *J. Chem. Soc.*, 1898). Rapid but not laborious exercise (on a bicycle) produces an increase in urea excretion, reaching a maximum in twelve hours, but not regaining the normal level for thirty hours afterward, the increase beginning immediately after the exercise. If the subject is not in good condition or the food insufficient, the rise is greater and lasts longer. There is an increase in the acidity after exercise which runs parallel to the urea, and a small increase in the urinary phosphates which follows the same course. The increase in the sulphates is proportional to that of the urea, but is of less duration and therefore of more intensity; it begins during the exercise, reaches a maximum in six hours or less, and terminates within twelve or fourteen hours. The chlorides of the urine diminish and vary with the amount of sweating. Turkish baths produce a reduction in the water and the chlorides excreted by the kidneys.

### SUGAR AS A FOOD.

Auguste Chauveau (*Compt. rend.*, 1898; *J. Chem. Soc.*, 1898). The general conclusions arrived at in this paper are as follows: The quantities of sugar, or of fat, that it is necessary to add to a given ration of meat in order to obtain the best diet for a man in work are not isodynamic quantities. An energy value of 0.756 in sugar is generally as effective as an energy value of 1.0 in form of fat, and under conditions the advantage of sugar may be still greater. In the case of sugar, the ratio of nutritive value to energy value is not constant, but may increase considerably when new tissues are being formed or an exhausted organism is being revivified, whereas in the case of fat the ratio remains practically constant. The increase in the relative nutritive value of the sugar is due to the fact that it promotes assimilation of proteids and reduces dissimilation. It follows that if it is misleading to reduce the nutritive value of a food simply from its heat of combustion, it is equally wrong to deduce this value exclusively from the facility with which the food is converted into muscular glycogen. As a matter of fact, the nutritive value of a food depends not only on the energy that it is capable of supplying but also on the indirect influence that it is capable of exerting in the renewal and formation of the anatomical elements of the body.

From whatever point of view the matter is regarded, however, the superiority of sugar over fat is very distinct.

The author has reason to believe that these conclusions apply in the case of men at rest as well as in the case of men at work.

---

ABSORPTION OF IRON IN MAN.

G. HONIGMANN (*Virchow's Archiv.*, 1898; *J. Chem. Soc.*, 1898). Hoffmann's experiments on animals prove that absorption of inorganic preparations of iron takes place in the intestine. The present observations on a patient with a fistula in the lower ileum, and in whom the large intestine was thus rendered functionless, show, from examination of the intestinal contents during the administration of *ferrum citricum oxidatum*, that Bunge's hypothesis has no foundation, and that the alimentary canal, as far as the ileum has the power to absorb as much, is 0.33 gram of iron in two days. The urine only contained traces of iron.

---

ESTIMATION OF INDICAN IN URINE.

AMANU (*J. Chem. Soc.*, 1898). Twenty c.c. of urine is gently shaken with a few drops of sulphuric acid, 5 c.c. of chloroform and 5 c.c. of a 10 per cent. solution of sodium per sulphate; the indigo thus generated dissolves in the chloroform, coloring it a more or less deep blue.

This reaction is more delicate than the oxidation by means of a hypochlorite, hypobromite, or permanganate, and is not interfered with by the presence of albumin. Scatole gives a reddish violet coloration with the reagent, but the coloring matter does not pass into solution in the chloroform; the intensity of the color corresponds with the amount of scatole.

---

NEW METHOD OF DISINFECTION.

R. WALTHER and A. SCHLOSSMAN, (*Jour. Pr. Chem.*, 1898; *Jour. Chem. Soc.*, 1898,) after a lengthy review of all the known methods of disinfection, the authors state that by far the best results are obtained by using a solution of formaldehyde in glycerine, which they call "glycoformal." This is dispersed through the room to be disinfected in the form of a very fine mist, half an hour sufficing to absolutely destroy all germs.

